# Mismatch repair deficiency testing in Lynch syndrome-associated urothelial tumors

**DOI:** 10.3389/fonc.2023.1147591

**Published:** 2023-04-18

**Authors:** Maria Rasmussen, Peter Sowter, Richard Gallon, Jon Ambæk Durhuus, Christine Hayes, Ove Andersen, Mef Nilbert, Lone Schejbel, Estrid Høgdall, Mauro Santibanez-Koref, Michael S. Jackson, John Burn, Christina Therkildsen

**Affiliations:** ^1^ Department of Clinical Research, Copenhagen University Hospital - Amager and Hvidovre, Copenhagen, Denmark; ^2^ Translational and Clinical Research Institute, Faculty of Medical Sciences, Newcastle University, Newcastle upon Tyne, United Kingdom; ^3^ Center for Healthy Aging, Department of Cellular and Molecular Medicine, University of Copenhagen, Copenhagen, Denmark; ^4^ Institute of Clinical Sciences, Division of Oncology and Pathology, Lund University, Lund, Sweden; ^5^ Molecular Unit, Department of Pathology, Copenhagen University Hospital – Herlev and Gentofte, Copenhagen, Denmark; ^6^ Biosciences Institute, Faculty of Medical Sciences, Newcastle University, Newcastle upon Tyne, United Kingdom; ^7^ The Danish HNPCC Register, Gastro Unit, Copenhagen University Hospital - Amager and Hvidovre, Copenhagen, Denmark

**Keywords:** Lynch syndrome, urothelial cancer, universal testing, mismatch repair deficiency, immunohistochemistry, microsatellite instability

## Abstract

**Introduction:**

Lynch syndrome-associated cancer develops due to germline pathogenic variants in one of the mismatch repair (MMR) genes, *MLH1*, *MSH2*, *MSH6* or *PMS2*. Somatic second hits in tumors cause MMR deficiency, testing for which is used to screen for Lynch syndrome in colorectal cancer and to guide selection for immunotherapy. Both MMR protein immunohistochemistry and microsatellite instability (MSI) analysis can be used. However, concordance between methods can vary for different tumor types. Therefore, we aimed to compare methods of MMR deficiency testing in Lynch syndrome-associated urothelial cancers.

**Methods:**

Ninety-seven urothelial (61 upper tract and 28 bladder) tumors diagnosed from 1980 to 2017 in carriers of Lynch syndrome-associated pathogenic MMR variants and their first-degree relatives (FDR) were analyzed by MMR protein immunohistochemistry, the MSI Analysis System v1.2 (Promega), and an amplicon sequencing-based MSI assay. Two sets of MSI markers were used in sequencing-based MSI analysis: a panel of 24 and 54 markers developed for colorectal cancer and blood MSI analysis, respectively.

**Results:**

Among the 97 urothelial tumors, 86 (88.7%) showed immunohistochemical MMR loss and 68 were successfully analyzed by the Promega MSI assay, of which 48 (70.6%) were MSI-high and 20 (29.4%) were MSI-low/microsatellite stable. Seventy-two samples had sufficient DNA for the sequencing-based MSI assay, of which 55 (76.4%) and 61 (84.7%) scored as MSI-high using the 24-marker and 54-marker panels, respectively. The concordance between the MSI assays and immunohistochemistry was 70.6% (p = 0.003), 87.5% (p = 0.039), and 90.3% (p = 1.00) for the Promega assay, the 24-marker assay, and the 54-marker assay, respectively. Of the 11 tumors with retained MMR protein expression, four were MSI-low/MSI-high or MSI-high by the Promega assay or one of the sequencing-based assays.

**Conclusion:**

Our results show that Lynch syndrome-associated urothelial cancers frequently had loss of MMR protein expression. The Promega MSI assay was significantly less sensitive, but the 54-marker sequencing-based MSI analysis showed no significant difference compared to immunohistochemistry. Data from this study alongside previous studies, suggest that universal MMR deficiency testing of newly diagnosed urothelial cancers, using immunohistochemistry and/or sequencing-based MSI analysis of sensitive markers, offer a potentially useful approach to identification of Lynch syndrome cases.

## 1 Introduction

The cancer-predisposition syndrome, Lynch syndrome, is caused by pathogenic germline variants in the mismatch repair (MMR) genes *MLH1*, *MSH2*, *MSH6*¸ or *PMS2*. Beside a high risk of colorectal and endometrial cancer, Lynch syndrome has been linked to an increased risk of both upper (ureteral and renal pelvic) and lower (bladder) urothelial cancer (1–4), with urothelial cancers being the third most common cancer in this population. The highest risk of urothelial cancer is observed in patients with pathogenic germline variants in *MSH2* with cumulative risk estimates at age 70 of 5.8–6.9% for upper urinary tract (ureter and renal pelvis) cancer and 2.6–12.3% for bladder cancer, compared to 2.2–4.8% and 0–10.8% for those harboring *MLH1* pathogenic variants and 0–2.9% and 0–1.7% for those harboring *MSH6* pathogenic variants for upper urinary tract and bladder cancers, respectively (2, 4). The cumulative risk of urothelial cancer at age 70, can be as high as 25.5% for men (4).

Loss of MMR function is an early event in most Lynch syndrome tumors as a single hit in the still functioning MMR allele can induce total loss of function. The resulting MMR deficiency causes an excessive accumulation of mutations especially in small repetitive DNA sequences, referred to as microsatellite instability (MSI). As loss of MMR protein expression and/or MSI are found in most Lynch syndrome-associated cancers, these analyses can be used as screening tools to increase identification of Lynch syndrome individuals and facilitate cancer-preventive surveillance strategies for affected patients and their family members. In addition, loss of MMR protein expression and MSI analyses have gained increased clinical interest during recent years, as these biomarkers were approved by the American Food and Drug Administration (FDA) in 2017 to guide immunotherapy with pembrolizumab across tumor types (5, 6).

MMR deficiency in tumors can for most cases be visualized by either immunohistochemical staining using antibodies directed against the four MMR proteins to show loss of expression, or by MSI analyses that assess changes in the length of microsatellites by PCR and fragment length analysis (typically capillary electrophoresis) or sequencing-based methods (7–10). Immunohistochemical analyses can be difficult to interpret due to intra- and inter-observer variability or lack of internal positive control cells and they are insensitive to MMR missense variants that disrupt function whilst retaining protein expression. Therefore, a combination of the two tools has been proposed (11–13). A standardized panel of five MSI markers (BAT-25, BAT-26, NR-21, NR-24, and MONO-27), which comprise the MSI Analysis System v1.2 (Promega), has been widely used during the last two decades towards identification of Lynch syndrome colorectal cancer patients (14). However, the Promega MSI assay may be less sensitive for MMR deficiency than immunohistochemistry when used in extra-colorectal cancers (15, 16). Furthermore, this method classifies tumors into three disjunct categories: MSI-high (MSH-H), MSI-low (MSI-L), and microsatellite stable (MSS), where MSI-H indicates MMR deficiency. However, MSI classification can be sensitive to the number and identity of MSI markers investigated, and the biological reality is likely a gradient from MSS to MSI-H rather than categorical subsets (17–19).

New methods to assess MSI have consequently been developed that use alternative methods, markers, and automated and dichotomized classifiers (20–24). For example, the Idylla™ MSI Test uses PCR and high resolution melt curve analysis of seven alternative markers, and has shown promising results for colorectal cancer, though the concordance with immunohistochemical analyses was lower for some endometrial cancers (25, 26). Assessment of MSI status using next generation sequencing of microsatellites captured within targeted panel, exome, or genome sequencing can be achieved using a variety of classifiers. Three such classifiers, MSIsensor, mSINGS, and MANTIS, were tested in six types of cancer, including colorectal, endometrial, esophagus, gastric, and prostate cancers, and achieved a concordance of 77%–100% when compared to the Bethesda MSI assay using PCR and fragment length analyses of three dinucleotide and two mononucleotide markers (20). Unfortunately, no urothelial tumors were analyzed in this study.

We have previously tested the Promega MSI assay in a small cohort of Lynch syndrome-associated urothelial cancers and found only 23% of the tumors to be MSI-H while 90% had immunohistochemical loss of MMR proteins (2), suggesting that MSI analysis of urothelial cancers may not be a suitable screen for Lynch syndrome. A gene panel sequencing-based analysis that classified 551 unselected urothelial cancers using MSIsensor found that 5.8% had increased MSI, and that 37.5% of these were Lynch syndrome-associated (27), demonstrating the potential clinical utility of a different MSI analysis method. This study was, however, conducted in an anonymized cohort, and immunohistochemical analyses of the MSS Lynch syndrome-associated cancers were not possible, meaning that false-negatives may have been missed and concordance between these two methods was not established. Hence, additional studies for immunohistochemical and MSI analyses for urothelial cancers are warranted.

A single molecule molecular inversion probe (smMIP), amplicon sequencing-based MSI assay of 24-markers previously achieved 100% sensitivity and 100% specificity in over 200 colorectal cancers compared to the Promega MSI assay (28, 29). Very recently, this assay has been further enhanced using a panel of 54 markers selected for instability in the normal peripheral blood of patients with the childhood cancer syndrome constitutional MMR deficiency (CMMRD). These novel 54 markers allowed much greater separation of CMMRD blood samples from controls than was achieved with the original 24 markers, and similarly improved MSI classification of colorectal cancers (30), indicating they may have utility in other tumor types as well. Here, we compare immunohistochemical MMR loss in an updated cohort of 97 Lynch syndrome-associated urothelial tumors to a variety of MSI assays including the Promega MSI Analysis v1.2 and the smMIP amplicon sequencing-based approach using both the 24-marker and 54-marker panels.

## 2 Materials and methods

### 2.1 Study population and samples

Individuals with surgically removed urothelial (ureteral, renal pelvic and/or bladder) cancer diagnosed between January 1st, 1980, to December 19th, 2017, with either a verified pathogenic germline variant in one of the MMR genes, *MLH1*, *MSH2*, *MSH6*, and *PMS2*, or being a first-degree relative (FDR) to such were included. The individuals were identified through the Danish Hereditary Non-Polyposis Colorectal Cancer (HNPCC) Register, and formalin-fixed paraffin-embedded tumor tissues were identified and collected from Pathology Departments around Denmark. Thirty-six of the cases have been investigated in a previous study (2).

Clinical data corresponding to the collected tumor specimen including genetic MMR variant, family relation, sex, tumor site, age at diagnosis, surgery date, tumor stage and differentiation grade were extracted from the HNPCC register.

The study was approved by the Scientific and Ethics Committee of the Capital Region of Copenhagen, Denmark (H-17001916) and the Data Protection Agency (AHH-2017-071).

### 2.2 Immunohistochemical staining of mismatch repair proteins

Immunohistochemical analyses of the MMR proteins on whole slides were available for 36 of the tumors and have been previously published (2). The remaining tumors (N=61) were analyzed at the Department of Pathology, Herlev Hospital, with ready-to-use antibodies against MLH1 (clone ES05, Agilent, California, USA), MSH2 (clone FE11, Agilent), MSH6 (clone EP49, Agilent), and PMS2 (clone EP51, Agilent) using the Dako PT link machine (Agilent) with high pH antigen retrieval buffer for pretreatment and the Dako Omnis (Agilent) and the EnVision FLEX, high pH, kit (Agilent) according to manufacturer’s instructions. The immunohistochemical analyses were performed on tissue microarrays (containing 2 × 1 mm biopsies from tumor areas located using Hematoxylin and Eosin whole slide staining of each tumor) or 4 µm thin slices for 8 of the tumors.

All immunohistochemical stained slides were scanned and evaluated using the NDP.view2 viewing software (Hamamatsu Photonics K.K., Shizuoka, Japan) by two independent observers (MR and CT). Loss of MLH1, MSH2, MSH6, and PMS2 was defined as protein expression in ≤10% of the tumor cells in the presence of internal positive staining in control cells e.g., lymphocytes or stromal cells. This arbitrary cut-off was used as immunohistochemical screening of colorectal and endometrial cancer has shown that half of the cases with <10% positive tumor cells can be explained by pathogenic MMR germline mutations (31). This approach does not consider subclonal loss of MMR expression among tumor cells. We did, however, not find subclonal MMR loss in any of the samples studied. Only one sample was difficult to score with 30% positive tumor cells. This tumor was scored as positive according to the scoring criteria above but is mentioned in detail in the results.

### 2.3 DNA extraction

DNA was extracted from formalin-fixated paraffin-embedded tumor samples using the QIAamp^®^ DNA FFPE Tissue kit (QIAGEN, Germany). Samples were either processed as 10 µm sections, macro-dissected sections, or cores (1 mm in diameter), depending on the amount of tumor cells within the block and the thickness of the block. Sections and macro-dissected samples were incubated in 320 µL deparaffinization solution (QIAGEN), vortexed, incubated at 56°C for 3 minutes, cooled to room temperature (RT) and centrifuged at 11,000 x g for 1 minute. However, core samples were treated twice with 1ml xylene (Histolab, Sweden) for 30-60 minutes and centrifuged for 2 minutes at max speed in between and after. Pellet was resuspended in 99.5% ethanol and incubated for 30-60 minutes, supernatant was removed, and pellet was dried for 20 minutes. 180 µL ATL buffer (QIAGEN) was added, and the tubes were centrifuged at 11,000 x g for 1 minute. Subsequently, 20 µL proteinase K (QIAGEN) was added to the clear phase and incubated at 56°C overnight on an orbital shaker at 650 RPM. If lysis was not complete, additional 20 µL proteinase K was added. The samples were then incubated at 90°C for a maximum of 60 minutes, centrifuged, and the clear phase was transferred to new tubes to which 2 units of AmpErase™ uracil N-glycosylase (UNG) (Thermo Fisher Scientific, Massachusetts, USA) was added. Samples were vortexed, centrifuged, and incubated at 50°C for 60 minutes, and subsequently centrifuged and incubated for 2 minutes at room temperature with 2 µL RNase A (QIAGEN). The rest of the DNA extraction was carried out on an automated QiaCube (QIAGEN) platform as described by the manufacturer and eluted in 40 µL distilled water.

DNA concentrations were measured using Qubit dsDNA BR kit and fluorometer 3.0 (Invitrogen, Massachusetts, USA) to determine the volume needed for the MSI analyses. Only samples with double stranded DNA concentrations above 1 ng/μL could be analyzed for MSI-status, resulting in exclusion of 2 samples.

### 2.4 Microsatellite analysis using Promega MSI analysis system version 1.2

MSI analysis using the Promega MSI Analysis System v1.2, including mononucleotide repeat markers *BAT-25*, *BAT-26*, *NR-21*, *NR-24*, and *MONO-27* plus two control pentanucleotide repeat markers to distinguish between samples and detect possible contamination (14). This analysis was performed according to the manufacturer’s protocol, however without use of matched normal DNA. PCR amplification used a Verity 96 thermocycler (Applied Biosystem, Massachusetts, USA). DNA fragments were separated by capillary electrophoresis on the 3130*xl* Genetic Analyzer (Applied Biosystem, Massachusetts, USA) and data was analyzed with GeneMapper Software 5 (Thermo Fisher Scientific, Massachusetts, USA) using AFLP default analysis settings. Interpretation was done by manual visual inspection of electropherogram traces by two experts in molecular biology (LS and EH). Due to the lack of matched normal samples, only samples with three or more unstable markers were classified as MSI-H (32). Samples with no unstable markers were classified as MSS. Samples with one unstable marker were classified as unresolved MSS/MSI-L, while samples with two unstable markers were classified as unresolved MSI-L/MSI-H. Samples with one or more markers with traces suggestive of instability that could not be confirmed due to lack of paired normal tissue were considered non-evaluable. To evaluate concordance with immunohistochemistry, MSI-L/MSI-H was pooled with MSI-H and referred to as pooled MSI-H, while MSS/MSI-L was pooled with MSS and referred to as pooled MSS/MSI-L.

### 2.5 Sequencing-based microsatellite instability analysis

#### 2.5.1 MSI markers and probe pooling, and phosphorylation

The MSI markers used in the smMIP amplicon sequencing-based MSI approach were mononucleotide repeats selected in previously published studies. Two panels were tested: A panel of 24 mononucleotide repeats selected based on their instability in sequence data from colorectal cancers (28, 29), and a panel of 54 markers selected based on their instability in whole genome sequencing data of normal (non-neoplastic) blood from patients with CMMRD (30). smMIPs to capture the MSI markers are described in the above-mentioned studies. All the probes were pooled, 5’-phosphorylated, and diluted to 0.1 nM per probe as previously described (28, 33).

#### 2.5.2 Probe target capture and amplification

MSI markers were probe-captured and amplified in multiplex using the previously described protocol (28, 33) using 23-273ng of sample DNA and a SensoQuest thermocycler. In brief, the targeting arms of the probes were annealed to the sample DNA template, the gap between the arms filled by a high-fidelity polymerase, and the 3’ end ligated to the 5’ end of the probe, resulting in circularized products. Linear DNA (sample DNA and excess probes) was degraded by exonucleases. Finally, the circularized probes containing the regions of interest were amplified by conventional PCR using universal primers that amplify from the common probe backbone sequence. Amplicons were analyzed by capillary electrophoresis using a QIAxcel (Qiagen, Germany) and the AL420 program, expecting amplicons in the range of 222–287 base-pairs (bp).

#### 2.5.3 Library preparation and sequencing

Amplicons were purified by Agencourt AMPure XP beads (Beckman Coulter, Indianapolis, USA) and quantified by Qubit dsDNA HS Kit (Invitrogen) using a Qubit Fluorometer 3.0 (Invitrogen). Purified amplicons with a dsDNA concentration of at least 1.89 ng/μL (12nM) were diluted to 4 nM in 10 mM Tris, pH 8.5, and then pooled in equal volumes to create the sequencing library. The library was sequenced using a 12 pM loading concentration and MiSeq v3 Kit (Illumina, California, USA) on a MiSeq platform (Illumina) following manufacturer’s protocols. The Generate FASTQ workflow, paired‐end sequencing, and custom sequencing primers were used as previously described (28, 33) to a target depth of >2000 reads per amplicon per sample.

#### 2.5.4 Bioinformatic analysis and MSI classification

Microsatellite variant detection and MSI classification using the frequency and allelic bias of microsatellite deletions and a naïve Bayesian approach has been described previously (29). Published data from a cohort of 50 MSI-H and 52 MSS colorectal cancers analyzed by both the 24-marker and 54-marker assays and the same smMIP amplicon sequencing-based protocol (30) was used to train the MSI classifier. An MSI score <0 classified a sample as MSS, while a score >0 classified a sample as MSI-H.

### 2.6 Statistical analyses

Clinical data and results of MSI and immunohistochemical analyses were imported into R (34) in which all statistical analyses were performed. Comparison of the characteristics for carriers of germline pathogenic variants and FDRs were performed with Fisher’s exact test for categorical variables, while Welch Two Sample t-test was used to compare the mean age. Concordance between the immunohistochemical analysis and each MSI assay was calculated as the frequency of matched samples, by matching retained MMR protein expression with pooled MSS/MSI-L and loss of MMR protein expression with pooled MSI-H, divided by the total amount of successfully analyzed samples. Concordance was tested using the Exact McNemar test while the confidence intervals were calculated using Wilson confidence intervals. Concordance was visualized using ggplot2 package (35). Specificity and sensitivity for each method were calculated using the epiR package in R (36). The Exact McNemar test was also used to compare the two smMIP sequencing-based panels. To investigate the association between explanatory factors, such as tumor location, age of the archival material, MMR gene, DNA quantity, and whether a sample could be evaluated by MSI analysis, Fisher’s exact test was used for categorical variables while the Kruskal-Wallis Rank Sum Test was used for continuous variables. All statistical analyses were two-tailed and statistical significance was reached when p < 0.05. Truncating variants as defined here include frameshift, splice-site, large deletions, and non-sense variants.

## 3 Results

### 3.1 Cohort characteristics

In total, 97 tumors resected from verified carriers or FDRs of known carriers of germline pathogenic variants in *MSH2* (66.0%), *MSH6* (23.7%), *MLH1* (9.3%), or *PMS2* (1.1%), were retrospectively collected and analyzed (**Table 1**). The mean age at diagnosis was 63.9 years and the majority of the tumors developed in women (59.8%). The tumor location was equally distributed between bladder tumors (including one ureteric orifice) (37.1%), renal pelvic tumors (35.1%), and ureteral tumors (27.8%) (**Table 1**). *MSH2* was more frequently affected in carriers, while *MSH6* was almost as frequently affected as *MSH2* in FDRs (p < 0.001) (**Table 1**). There were no significant differences in sex, age, or tumor location between carriers and FDRs (**Table 1**). Ten of the included patients developed two or more synchronous tumors, while five patients developed metachronous tumors, and one developed both synchronous and metachronous tumors.

**Table 1:** Clinical data of Lynch syndrome-associated urothelial cancers.

Characteristics	All urothelial cancers	Cancers from carriers	Cancers from FDRs	P-value
**Age at diagnosis (range and mean)**	63.9 (31–89)	62.8 (42–82)	67.09 (31–89)	0.189
**Sex, N (%)**	**N=97**	**N=74**	**N=23**	0.089
Male	39 (40.2%)	26 (35.1%)	13 (56.5%)	
Female	58 (59.8%)	48 (64.9%)	10 (43.5%)	
**Genes, N (%)**	**N=97**	**N=74**	**N=23**	<0.001
*MLH1*	9 (9.3%)	7 (9.5%)	2 (8.7%)	
*MSH2*	64 (66.0%)	53 (71.6%)	11 (47.8%)	
*MSH6*	23 (23.7%)	14 (18.9%)	9 (39.1%)	
*PMS2*	1 (1.1%)	0 (0%)	1 (4.3%)	
**Location, N (%)**	**N=97**	**N=74**	**N=23**	0.912
Ureter	27 (27.8)	21 (28.4%)	6 (26.1%)	
Renal pelvis	34 (35.1)	25 (33.8%)	9 (39.1%)	
Bladder (including ureteric orifice)	36 (37.1)	28 (37.8%)	8 (34.8%)	

### 3.2 Loss of mismatch repair protein expression

Of the 97 tumors, 86 (88.7%) showed loss of MMR protein expression, all correlating to the MMR gene affected in the respective family (**Supplementary Table 1**). MMR protein loss was more frequent in carriers, with 69 out of 74 (93.2%) showing MMR protein loss, compared to 17 out of 23 (73.9%) FDRs (p = 0.020), suggesting that the FDR group may include non-carriers. The five MMR proficient tumors from carriers were observed in carriers of *MSH2* (N=1) and *MSH6* (N=4). One of the four *MSH6* variants was a missense variant (c.3259C>T), which has been associated with low levels of MSI, suggesting it might have lost MMR function while retaining MMR protein expression in a subset of the tumor cells (30%). We found a significant difference between upper and lower tract urothelial cancer and MMR protein expression, with nine of the 11 tumors with retained MMR protein expression being in the lower urothelial tract (p = 0.002) (**Table 2**). When dividing the cohort by carrier status, tumor location was significantly associated with immunohistochemical MMR protein expression only for the FDRs: Five of the six FDR tumors and four of the five carrier tumors with retained MMR protein expression were in the lower urinary tract (p = 0.009 and p = 0.065, respectively). This suggest that some of the lower urothelial tract cancers might be due to sporadic origin and not Lynch syndrome.

**Table 2:** Concordance between immunohistochemical analysis and the three MSI analyses divided by carrier status and tumor location.

		Total	Promega*	24-marker	54-marker
Carriers		N	N (%)	N (%)	N (%)
**Ureter/renal pelvis**	**MMR protein loss**	**45**			
	MSI		25 (56%)	30 (67%)	33 (73%)
	MSS		8 (18%)	5 (11%)	2 (4%)
	NE		12 (27%)	10 (22%)	10 (22%)
	**Retained MMR protein**	**1**			
	MSI		1 (100%)	0 (0%)	0 (0%)
	MSS		0 (0%)	1 (100%)	1 (100%)
	NE		0 (0%)	0 (0%)	0 (0%)
**Bladder**	**MMR protein loss**	**24**			
	MSI		13 (54%)	15 (63%)	15 (63%)
	MSS		5 (21%)	1 (4%)	1 (4%)
	NE		6 (25%)	8 (33%)	8 (33%)
	**Retained MMR protein**	**4**			
	MSI		1 (25%)	1 (25%)	2 (50%)
	MSS		1 (25%)	3 (75%)	2 (50%)
	NE		2 (50%)	0 (0%)	0 (0%)
FDR		N	N (%)	N (%)	N (%)
**Ureter/renal pelvis**	**MMR protein loss**	**14**			
	MSI		6 (43%)	8 (57%)	9 (64%)
	MSS		3 (21%)	2 (14%)	1 (7%)
	NE		5 (36%)	4 (29%)	5 (36%)
	**Retained MMR protein**	**1**			
	MSI		0 (0%)	0 (0%)	0 (0%)
	MSS		0 (0%)	1 (100%)	1 (100%)
	NE		1 (100%)	0 (0%)	0 (0%)
**Bladder**	**MMR protein loss**	**3**			
	MSI		1 (33%)	1 (33%)	1 (33%)
	MSS		1 (33%)	0 (0%)	0 (0%)
	NE		1 (33%)	2 (67%)	2 (67%)
	**Retained MMR protein**	**5**			
	MSI		1 (20%)	1 (20%)	1 (20%)
	MSS		2 (40%)	4 (80%)	3 (60%)
	NE		2 (40%)	1 (20%)	1 (20%)

*For readability the Promega assay score “MSI-L/MSI-H” was categorized as “MSI”, while “MSS/MSI-L” was categorized as “MSS”.

### 3.3 Microsatellite instability analysis using promega MSI analysis system v1.2 and concordance with immunohistochemistry

**Figure 1:**
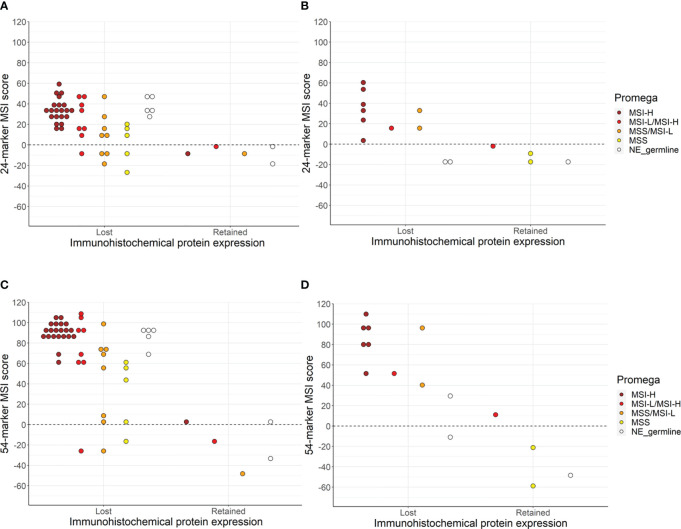
The concordance between immunohistochemical protein expression, Promega MSI assay, and sequenced-based MSI assay using the 24-marker assay for carriers **(A)**, and FDRs **(B)** or the 54-marker assay for carriers **(C)** and FDRs **(D)**. NE_germline are the samples that were non-evaluable (NE) due to the lack of germline DNA.

The Promega assay gave interpretable results for 68 of the tumors (70.1%) of which 36 (52.9%) were MSI-H, 12 (17.6%) were MSI-L/MSI-H, 12 (17.6%) were MSS/MSI-L, and eight (11.8%) were MSS (**Supplementary Table 1**). Immunohistochemical MMR protein expression and Promega MSI analyses were concordant for 48 of the samples (70.6%) (95% CI 58.8–80.1%, p = 0.003) (**Figure 1**). Assuming that the immunohistochemical analysis is the reference method for identification of cases with underlying germline pathogenic MMR variants, the Promega MSI assay could identify Lynch syndrome cases with a sensitivity of 72.6% (95% CI 59.8–83.1%) and a specificity of 50.0% (95% CI 11.8–88.2%).

Investigating the cases with retained MMR protein expression and MSI status, we found three tumors with retained expression that were MSI-H (N=1) or MSI-L/MSI-H (N=2) (**Supplementary Table 1**, **Table 2**). A possible explanation for this discordance could be missense variants, leading to a non-functional MMR protein but retained MMR protein expression. However, all three cases (two carriers and one FDR) were associated with germline truncating variants.

Investigating the MSS cases with loss of immunohistochemical MMR protein expression, we found 17 samples that were MSS or MSS/MSI-L and had loss of immunohistochemical expression of MMR proteins. Thirteen of these were from carriers, including one carrier of a truncating variant in *MLH1*, seven carriers of a variant in *MSH2* with six known to be truncating and the seventh not having a type reported, and five carriers of truncating variants in *MSH6* (**Supplementary Table 1**, **Table 2**). For the Promega MSI analyses, there was no statistically significant correlation between MSI status (using pooled classification, see Materials and methods) and tumor location (p = 0.415).

### 3.4 Microsatellite instability analysis using smMIP amplicon sequencing

The smMIP amplicon sequencing-based MSI assay was successfully performed for 72 of the included tumors (75.6%) of which 62 (86.1%) had loss of MMR protein expression. The 24-marker assay classified 55 of the tumors as MSI-H (76.4%), while the 54-marker assay identified six additional tumors as MSI-H (N=61, 84.7%) (p = 0.031) (**Figure 1**). A comparison between the 24-marker assay and immunohistochemical MMR protein expression showed that all but one of the 55 tumors that were MSI-H had loss of MMR protein expression, while nine out of the 17 MSS tumors had retained MMR protein expression (**Supplementary Table 1**, **Table 2**). This gave a concordance of 87.5% (95% CI 77.6–93.4%, p = 0.039), sensitivity of 87.1% (95% CI 76.1–94.3%), and specificity of 90% (95% CI 55.5–99.7%) for the 24-marker assay compared to immunohistochemical analyses. The one tumor with retained MMR protein expression that was MSI-H was from a carrier with a missense *MSH6* variant (c.3259C>T), which showed loss of MSH6 protein expression in 70% of the tumor cells. This sample was not evaluable in the Promega assay due to lack of germline DNA. The eight MSS tumors with immunohistochemical protein loss developed in six carriers and two FDRs. Of the six carriers, one had a truncating *MSH2* variant, while the remaining five had truncating *MSH6* variants. There was no significant association between MSI status and upper versus lower urothelial cancer (p = 0.253).

For the new 54-marker panel, three of the 61 MSI-H tumors had retained MMR protein expression, while seven of the 11 MSS samples showed retained MMR protein expression (**Supplementary Table 1**, **Table 2**). This gave a concordance of 90.3% (95% CI 80.9–95.4%, p = 1), a sensitivity of 93.5% (95% CI 84.3–98.2%), and a specificity of 70% (95% CI 34.8–93.3%) for the 54-marker sequencing-based MSI assay relative to immunohistochemical analysis. Retained MMR protein expression was found for three MSI-H samples, in an *MSH6* carrier with a truncating variant, an *MSH6* carrier with a missense variant (this was the same tumors as for the 24-marker assay), and an FDR with a truncating *MSH2* variant. Of the 11 MSS tumors, three carriers and one FDR had loss of MMR protein expression. Of the carriers, two had a truncating *MSH6* variant and one had a truncating *MSH2* variant. Again, there was no significant association between MSI status and tumor location (p = 0.173). Overall, for the 54-marker assay, an increase in the separation of the sample scores was seen compared to the 24-marker assay with a mean score of 58.6 (range -58.9–109.9) compared to 19.9 (range -26.9–60.4).

### 3.5 Discordance between the two sequencing-based assays

Of the six samples classified as MSS by the 24-marker panel but MSI-H by the 54-marker panel, five had low positive scores ranging from 0.7–11.1 (**Table 3**). Four belonged to carriers, all having truncating *MSH6* variants. One of these had retained expression of MSH6 but was MSI-H when analyzed by the Promega MSI assay. The three remaining *MSH6* carriers all had loss of MSH6 protein expression, and one was MSS and two were MSS/MSI-L when analyzed by the Promega assay. The remaining two cases developed in FDRs, one belonging to a family with a truncating variant in *MSH2* and this individual’s tumor had retained MSH2 expression but was MSI-L/MSI-H when analyzed by the Promega assay. The other FDR belonged to a family with a truncating *MSH6* variant, and the tumor showed loss of expression of MSH6. The Promega data was inconclusive due to missing germline DNA. This sample had the largest change in MSI score, from -17.6 for the 24-marker assay to 29.5 for the 54-marker assay. Together, these observations suggest the 54-marker assay may be more sensitive than the 24-marker assay.

**Table 3:** Overview of the six samples that were discordant with the 24- and 54- marker panels.

Carrier status	Gene	MMR protein immunohistochemistry	Promega MSI assay classification	24-marker assay MSI score	54-marker assay MSI score
FDR	MSH2	Retained	MSI-L/MSI-H	-1.9 (MSS)	11.1 (MSI-H)
Carrier	MSH6	Retained	MSI-H	-7.3 (MSS)	3.8 (MSI-H)
FDR	MSH6	Lost	NE_germline	-17.6 (MSS)	29.5 (MSI-H)
Carrier	MSH6	Lost	MSS/MSI-L	-10.3 (MSS)	4.7 (MSI-H)
Carrier	MSH6	Lost	MSS/MSI-L	-7.7 (MSS)	8.7 (MSI-H)
Carrier	MSH6	Lost	MSS	-26.9 (MSS)	0.7 (MSI-H)

### 3.6 Non-evaluable samples for the MSI assays

Twenty-nine tumors were non-evaluable by the Promega MSI assay due to technical issues such as low signal intensity or failed amplification (N=18), or due to uncertain results that could not be determined without germline material for comparison (N=11). Samples being non-evaluable due to technical issues was significantly associated with the age of the tumor blocks (p = 0.004), but not with DNA concentration (p = 0.644). For the sequencing-based assays, 25 samples were excluded from sequencing, fourteen of which were also non-evaluable by the Promega assay. The excluded samples were again from significantly older tumor blocks (p < 0.001) but were not associated with DNA concentration (p = 0.476).

## 4 Discussion

Testing for tumor MMR deficiency has dual clinical functions - both as a screening tool to identify Lynch syndrome patients and to guide use of immune checkpoint blockade therapy, especially within colorectal cancer. Both immunohistochemical protein expression and MSI analyses can be used to investigate MMR deficiency. However, these methods have primarily been developed for and used in colorectal cancers. Limited data is available for extra-colonic cancers, including urothelial cancers, especially comparing the two methods. Molecular studies investigating urothelial cancer primarily aim to screen for Lynch syndrome patients in large, unselected cohorts using immunohistochemistry and/or MSI analysis and germline MMR test a selected subgroup. Thus only few Lynch syndrome cases are included and often Lynch syndrome tumors with retained MMR protein and/MSS tumors will not be MMR germline tested (37, 38). Hence, the proportion of Lynch syndrome patients with retained immunohistochemical MMR protein expression and/or MSS status remains uncertain. In this study, we tested a large and updated cohort of 97 Lynch syndrome-associated urothelial cancer, to our knowledge the largest cohort of its type to be tested for MMR deficiency. Using immunohistochemical analyses of MMR protein expression, we found 88.7% had lost MMR protein expression in accordance with the gene affected within the family. This is in accordance with smaller studies of Lynch syndrome-associated urothelial cancer, in which 82-100% of the tumors showed loss of MMR protein expression (4, 39). In contrast, a previous study of upper tract urothelial cancer, reported loss of immunohistochemical MMR protein expression in 30% of the patients with a common Lynch syndrome-associated cancer, including patients with no verified Lynch syndrome-associated pathogenic MMR variant, and it was not possible to extract the numbers from verified Lynch syndrome individuals only (40). This study also included FDRs who might be non-carriers, explaining some of the tumors without loss of MMR protein expression. During the final preparation of this manuscript, one of the included FDRs was found to be a non-carrier from a recent gene test. This individual was, however, kept in the study as molecular and statistical analyses had already been performed and since MMR proficient samples are valuable for the concordance analyses and are scarce in the study cohort. Considering only carriers, 93.2% had immunohistochemical loss of MMR protein expression. This correlates well with what previous studies found for other Lynch syndrome-associated extra-colorectal cancers (41–44). In summary, these data indicate that immunohistochemical MMR protein expression can be used to identify Lynch syndrome cancers but does not identify all. We found an association between retained MMR protein expression and lower urinary tract cancers, which might be explained by lower tract urinary cancers are more common in the general population than upper tract urinary cancers and Lynch syndrome individuals can also develop sporadic cancers, i.e., not due to their underlying MMR germline variant (45). MMR immunohistochemistry has limitations since missense MMR variants might not be identified with immunohistochemistry and the evaluations are subjective (11,1213). Hence, objective evaluations, also giving a dichotomized evaluation, could be warranted.

We have previously analyzed a small cohort of Lynch syndrome urothelial cancer and shown that the conventional Promega MSI method had difficulties identifying all the MMR protein deficient tumors (2) (36 of the tumors included in this study). In this updated and larger cohort, the Promega assay found 52.9% of the tumors to be MSI-H and 17.6% to be MSI-L/MSI-H (pooled MSI-H = 70.5%). We also tested a new sequencing-based method with two different MSI marker sets, one previously derived from colorectal cancer sequence data (28), and the other containing MSI markers derived from whole genome sequencing of normal (non-neoplastic) blood of individuals with CMMRD syndrome, which was found to be more sensitive than the 24-marker panel also for colorectal cancers (30). The 24-marker assay found 76.4% to be MSI-H, while the new 54-marker assay found 84.7% to be MSI-H. These results suggest that MSI analyses have potential to identify Lynch syndrome cancers, but careful marker selection and validation may be required.

For the 54-marker assay, we also observed a wider range of scores. This and its increased sensitivity can, in part, be explained by the larger number of markers analyzed compared to the 24-marker assay. However, in the study in which they were identified it was shown that these new MSI markers were individually more sensitive than the original 24-marker panel for both constitutional and tumor analyses (30). It was evident that five of the tumors, that were MSS in the original panel gave a low score above zero, since they are close to the threshold separating MSS and MSI-H. These five could potentially be false positives in the 54-marker assay. However, as four of these were from *MSH6* carriers and one FDR from an *MSH6* family this might explain the low scores, since they generally were lower for *MHS6* (30). If the samples were false negatives in the 24-marker assays this could be explained by subclonal MMR deficiency, where the MSI signal is just below the limit of detection. False negatives/positives could also be explained by the MSI classifier being trained using colorectal cancers and for future studies it would be pertinent to re-train the MSI classifier on urothelial cancers.

Difference in the MSI-H proportion have been observed for Lynch syndrome cancer depending on the specific organ. A previous study analyzing MSI in Lynch syndrome-associated tumors using the Bethesda MSI panel, which includes mononucleotide and dinucleotide repeat markers, found that most of the ureteral tumors were MSI-H whilst the frequency was lower for bladder cancer, kidney cancer, and brain cancer (39). Likewise, MSIsensor found varying frequencies of samples being MSI-H in different cancer types (27). We did not find a difference between upper and lower urothelial cancers and the MSI status for the three MSI assays, only for immunohistochemical MMR protein expression. This was only significant for the FDR cohort but not the carrier cohort, albeit a similar trend was observed. There was a correlation between lower tract urothelial tumors and retained MMR protein expression. This suggests that the tumors might be sporadic and not due to the underlying germline MMR variant. This is consistent with lower tract urothelial cancer being more common in the general population (45).

The concordance between the MSI assays and immunohistochemical MMR protein expression was highest for the 54-marker assay (90.3%) and lowest for the Promega assay (70.6%). A systematic review of universal screening of upper tract urothelial cancer, found a concordance of 58.7% (range 40%-100%) between immunohistochemical MMR protein expression and (primarily) the Bethesda panel (38). In total, 57 samples gave evaluable results by all three MSI assays, of which the sequencing-based method performed better than the Promega MSI assay. According to immunohistochemical MMR protein expression, only 39 tumors showed concordance using the Promega assay, while 51 samples were concordant using the 24-marker assay, and 52 tumors using the 54-marker assay. Exome and genome sequencing increase the potential number of MSI markers to analyze. However, increasing the number of MSI markers analyzed may not increase sensitivity and specificity as assay accuracy also depends on the sensitivity and specificity of the individual MSI markers. Indeed, assay sensitivity and specificity can be maintained or even improved with smaller, select MSI marker panels (20, 21, 28, 30). MSI and immunohistochemical MMR protein expression have been used interchangeably but based on this study the two types of analyses does not always identify the same samples. However, MSI can identify a few additional samples that does not show immunohistochemical loss so it could be used in addition to immunohistochemistry to identify as many tumors with MMR deficiency as possible. A recent review of the diagnostic yield of universal immunohistochemical testing has shown that this test identifies up to 4.7% individuals with Lynch syndrome (36). The screening was, however, designed in a way that only MMR deficient tumors were referred to MSI testing and/or genetic analyses, which results in the lack of identification of Lynch syndrome tumors that are MMR proficient but MSI.

In addition, Lynch syndrome-associated extra-colorectal cancers have been found to have a lower concordance between MSI and immunohistochemistry MMR protein expression. In a study of Lynch syndrome-associated ovarian cancer, six patients were tested for both immunohistochemical loss and Promega MSI with four being MSI-H giving a concordance of 66.7% (15). In a study in our group we found a concordance between Idylla™ MSI and immunohistochemical loss of 60.0% for Lynch syndrome-associated cancer (43), while concordance was 96.4% for immunohistochemical protein expression and Idylla™ MSI for colorectal cancer (46). For Lynch syndrome-associated endometrial cancer, immunohistochemical loss identified 100% while MSI-H with Promega were only identified in 56% of the investigated tissues (16). The lower concordance for extra-colorectal cancers, might also suggest that other markers than the ones used in the Promega assay might be more characteristic for MSI status in other tumor types. As mentioned above, MSI status was not significantly different between upper and lower tract urothelial cancers for any of the MSI assays used in this study. Furthermore, all of the sequencing-based markers were intergenic, whereas Promega had both intronic and exonic markers (47), which might affect their alterations differently depending on organ. Other reasons might be due to timing of MMR deficiency with immunohistochemical loss as an initial step followed by MSI at a later stage. This could be analyzed by TNM stage and MSI status as the number of altered microsatellite sequences are likely to increase during tumor development. However, we did not investigate this due to lack of complete TNM data.

The MSI status from both types of assays did not correlate completely with the immunohistochemical staining and we found tumors with retained MMR protein expression showing MSI. One reason for this, could be that retained immunohistochemical protein expression does not necessarily mean that the MMR-protein is functional. It has been found that Lynch syndrome-associated colorectal tumors express MMR proteins despite having a missense germline variant to a higher degree than truncating variants (11). These tumors have been found to be MSI-H (48, 49). In here, we found four tumors that were MSI-H but with retained MMR-proteins with either of the two types of assays but only one of these cases could be explained by a germline *MSH6* missense variant, in which 30% of the tumor cells expressed all MMR proteins. Additional reasons could be unknown genetic or epigenetic mutations affecting the MMR system such as the somatic second hit being a missense variant. In addition, it is well-known that immunohistochemical MMR protein expression analyses does not identify loss in all Lynch syndrome-associated tumors (7, 8, 50).

In this study, it was not possible to analyze all the samples for MSI, and of the 29 (29.9%) non-evaluable samples for the Promega assay and the 25 (25.8%) samples excluded from the sequenced-based MSI analyses, 14 (14.4%) could not be evaluated by either assay. Although formalin fixation and paraffin embedding is an efficient way to store tumor tissues it can lead to DNA degradation, hence, lower DNA quality and quantity, which will lead to PCR errors such as stutter bands in the electropherogram traces and less amplification product (51, 52). In this cohort 82.5% (N=80) of the tumors were surgically removed in 1980-2011, hence they are more than 10 years old. The non-evaluable samples were significantly older than the evaluable samples, but no significant difference was found regarding DNA concentration or MMR gene. In this study, all 97 samples gave evaluable results when analyzed with immunohistochemical MMR protein analyses; hence, this may be the best method in a retrospective setting. However, the two methods might complement each other.

In contrast to the Promega assay, the MSI classification used by the sequencing-based assay only focuses on deletions, since similar levels of insertions in mononucleotide repeats were found for both MSI and MSS samples but it differed for deletions (29). A limitation of the Promega assay, is the longer monomeric sequences as these may lead to PCR artifacts (53) that can affect the evaluation and thereby interpretation of MSI status. In addition, the fragment lengths are evaluated manually which may induce subjectivity. Our interpretation of Promega results were challenged by the lack of matched normal samples to check for germline polymorphisms and minor shifts in fragment lengths. Indeed, some of our urothelial cancer samples showed more subtle changes in mononucleotide lengths were found compared to the larger shifts we usually see for colorectal cancers, which has also been observed for endometrial cancer (54). This may lead to classification of fewer MSI-H cancers. To circumvent this, we subdivided the MSI groups even further, adding MSI-L/MSI-H and MSS/MSI-L, for the tumors that with two or one unstable markers, respectively. Of the 12 MSI-L/MSI-H tumors, nine were categorized as MSI-H using the 54-marker assay, indicating that inclusion of germline DNA might have improved the Promega results. In the new version of the Promega MSI kit, OncoMate™ MSI Dx Analysis System, recommendations for MSI analyses have been included and only deviations of three or more bp should now be considered MSI, just as it was stated previously by Suraweera (32). In addition, the new kit can also be used with automated software and these initiatives can lead to less subjective evaluations, although it still needs to be validated prior to implementation. The sequencing-based MSI classification used in this study is automatic, however, it is currently only trained for colorectal cancers which might lead to incorrect evaluation for other cancer types. Studies in a prospective setting are warranted and further validation is needed prior to clinical use.

Another consideration is the use of software that detect MSI and other mutational signatures of MMR deficiency alongside detection of MMR gene variants in gene panel, exome, and genome sequence data. Software like MMRDetect, MSIdetect, MSISensor, mSINGS, and MANTIS have demonstrated high accuracies (>95%) for the detection of MSI/MMR deficiency in a variety of tumor types (10, 20–22). Therefore, as these sequencing methods are increasingly used for diagnostic and treatment purposes, it is possible that targeted MSI analysis will become redundant. However, targeted MSI analysis is lower cost, generates significantly less data, and can allow faster turnaround times. Therefore, it will likely be a useful method for some time, particularly in low to middle-income countries.

## 5 Conclusion

We found that immunohistochemical analyses identified loss of MMR protein expression in 88.7% of the Lynch syndrome-associated urothelial tumors analyzed. Comparing the MSI assays with immunohistochemical analyses, Promega was concordant for 70.6%, the 24-marker assay for 87.5%, and the 54-marker assay for 90.3%. We did not find a statistical difference between immunohistochemistry and the 54-marker panel. However, as each method identified few unique MMR deficient cases, we recommend using both immunohistochemistry of MMR proteins and the 54-marker panel for a truly comprehensive test for both screening purposes and treatment with immune checkpoint inhibitors. This is a retrospective study performed on archival samples which should be validated in a prospective setting prior to clinical implementation.

## Data availability statement

The FASTQ files generated for the sequence-based MSI assay are available at the European Nucleotide Archive with the following study identification: PRJEB58093 (https://www.ebi.ac.uk/ena/browser/view/PRJEB58093). 

## Ethics statement

The studies involving human participants were reviewed and approved by Scientific and Ethics Committee of the Capital Region of Copenhagen, Denmark (HD-2007-0032 and H-17001916). Written informed consent for participation was not required for this study in accordance with the national legislation and the institutional requirements.

## Author contributions

Conceptualization: MR, PS, RG, MJ, JB, CT. Methodology: MR, PS, RG, JD, LS, EH, CH. Software: MR, PS, RG. Validation: MR, PS, RG, LS, EH. Formal analyses: MR, PS, RG, MS-K. Investigation: MR, PS, RG, CT. Resources: OA, MN, PS, EH, MJ, JB, CT. Data curation: MR. Writing – original draft: MR, CT. Writing – review and editing: MR, PS, RG, JD, CH, OA, MN, LS, EH, MS-K, MJ, JB, CT. Visualization: MR, RG. Supervision: RG, PS, MN, CT. Project administration: MR, PS, RG. Funding acquisition: MR, MN, CT. All authors contributed to the article and approved the submitted version.

## Supplementary material

Supplementary Table 1 An overview of the immunohistochemistry, MSI analyses and relevant clinical data.
